# Apport de l’échographie dans les traumatismes oculaires à Parakou (Bénin)

**DOI:** 10.11604/pamj.2013.15.114.2360

**Published:** 2013-07-28

**Authors:** Kofi-Mensa Savi de Tove, Abel Rodrigue Assavedo, Patricia Yekpe, Zakari Nikiema, Olivier Biaou, Vicentia Boco

**Affiliations:** 1 Département d’Imagerie Médicale, Faculté de Médecine, Université de Parakou, Bénin; 2 Département d’ophtalmologie, Faculté de Médecine, Université de Parakou, Bénin; 3 Département d’Imagerie Médicale, Faculté des Sciences de la Santé, Université d’Abomey-Calavi, Cotonou Bénin; 4 Service d’Imagerie Médicale, Centre Hospitalier Universitaire Sourô Sanou, Bobo-Dioulasso, Burkina-Faso

**Keywords:** Traumatisme oculaire, échographie, hémorragie vitréenne, décollement de la rétine

## Abstract

**Introduction:**

Décrire l’apport de l’échographie réalisée avec un appareil polyvalent dans les traumatismes oculaires.

**Méthodes:**

Il s’agit d’une étude prospective descriptive réalisée du 01 février au 01 aout 2010 dans le service d’imagerie médicale du CHD Borgou. Trente deux (32) patients présentant un traumatisme oculaire avec baisse de l’acuité visuelle ont bénéficié d’une échographie oculaire réalisée grâce à un échographe polyvalent.

**Résultats:**

L’âge moyen de nos patients était de 29,40 ans (extrêmes 1 et 68 ans). Le sexe ratio (H/F) était de 1,67.Sur 39 yeux traumatisés, l’il droit était atteint dans 22 cas (56,4%), et l’il gauche dans 17 (43,6%) cas. Les contusions ont constitué la variété anatomo-clinique la plus fréquente: 32 cas (82%). Les différentes lésions observées étaient: une hémorragie isolée du vitré 13 cas (33,3%), un décollement rétinien 6 cas (15,4%), un décollement choroïdien 6 cas (15,4%), un décollement postérieur du vitré 5 cas (12,8%), une cataracte 5 cas (12,8%) et une luxation postérieure du cristallin dans 3 cas (7,7%).

**Conclusion:**

L’échographie oculaire même réalisée avec un appareil polyvalent permet un bilan lésionnel satisfaisant des traumatismes oculaires.

## Introduction

Les traumatismes oculaires (TO) sont des affections relativement fréquentes avec des conséquences graves car pouvant entrainer une cécité. Leur prise en charge constitue une urgence aussi bien médicale que chirurgicale [1–3]. Cette prise en charge ne peut être efficiente sans un bilan lésionnel précis. Le fond d’œil est souvent peu contributif lorsqu’il existe une opacification des milieux transparents [4–7]. Dans ces cas, l’échographie oculaire qui est une technique simple, rapide, non agressive et de coût abordable permet l’exploration de l’œil. Toutefois indications sont multiples et comprennent, outre les inaccessibilités du fond d’œil (FO), la recherche de tumeurs oculaires, la biométrie et les traumatismes oculaires [8–10]. Cependant l’échographie oculaire reste de pratique peu courante au Centre Hospitalier départemental du Borgou (CHD-B), bien que cet hôpital dispose depuis plusieurs années, d’un échographe polyvalent. Les hypothèses pour justifier cette situation seraient que cet examen soit peu connu ou que ses apports au diagnostic soient sous évalués ou encore que peu de crédit soit accordé aux résultats fournis par un échographe polyvalent, seul disponible.

L’objectif de cette étude était de décrire l’apport diagnostic de l’échographie oculaire en mode B réalisée avec un échographe polyvalent au C.H.D du Borgou et de l’Alibori dans les traumatismes oculaires.

## Méthodes

Il s’est agi d’une étude prospective, transversale et descriptive. Elle s’était déroulée sur une période de six mois allant du 1er février au 1er aout 2010. Cette étude a été réalisée dans les services d’imagerie médicale et d’ophtalmologie du CHD-B. Ont été inclus, tous les patients qui ont consulté pour un traumatisme oculaire avec une baisse de l’acuité visuelle et chez qui une échographie oculaire a été réalisée.

Cette étude a obtenu l’aval du comité local d’éthique et le consentement verbal éclairé de tous les patients ou de leurs parents lorsqu’ils étaient mineurs.

Chaque patient a bénéficié d’un examen ophtalmologique réalisé par le même médecin ophtalmologiste. Cet examen comprenait la prise de l’acuité visuelle de loin et de près, l’examen au biomicroscope et la réalisation d’un examen du fond d’œil (FO) en l’absence d’une opacification des milieux transparents de l’œil. Les échographies ont été réalisées par un médecin radiologue ignorant les résultats de l’examen ophtalmologique grâce à un appareil polyvalent muni d’une sonde linéaire à fréquence variable (5-10MHz). L’examen était réalisé sur un patient en décubitus dorsal, le regard en position primaire, paupières closes. Des coupes axiales, transvitréennes et obliques sagittales ont été réalisées pour explorer successivement la chambre antérieure, le cristallin, le corps vitré, la rétine et le nerf optique. L’examen échographique était réalisé en modulant régulièrement la puissance du faisceau ultrasonore. Le vitré et la hyaloïde ont donc été examinés à gain élevé. La paroi, la rétine, et la choroïde ont été explorées à gain modéré. En l’absence d’anomalie à l’exploration échographique réalisée moins six heures après le traumatisme, une deuxième échographie est pratiquée le lendemain.

Les variables étudiées ont été les suivantes: l’âge, le sexe; le diagnostic clinique et les anomalies échographiques. L’œil a été considéré comme unité statistique. L’analyse a été descriptive et les données ont été traitées grâce au logiciel SPSS 10.

## Résultats

Notre série de 32 patients était constituée de 20 hommes et 12 femmes soit un sexe ratio (H/F) de 1,67. L’âge moyen de nos patients était de 29,40 ± 15,13 ans avec des extrêmes de 1 et 68 ans.

Les agressions étaient les circonstances de traumatisme les plus fréquentes avec 19 cas (59,4%) suivies des accidents de la voie publique (AVP) 8 cas (25%), des accidents de travail 3 cas (9,4%) et des accidents de sport 2 cas (6,2%).

Le traumatisme était unilatéral dans 25 cas (78,1%) et bilatéral dans 7 cas (21,9%). Le nombre d’yeux traumatisés était de 39. L’œil droit était atteint dans 22 cas (56,4%).

Les contusions ont constitué la variété anatomo-clinique la plus fréquente trouvée dans 32 cas (82%). Les plaies du globe étaient présentes dans 7 cas (18%). La contusion était unilatérale dans 19 cas et bilatérale dans 6 cas. Les plaies étaient unilatérales dans 6 cas et bilatérales dans 1 cas.

**Figure 1:**
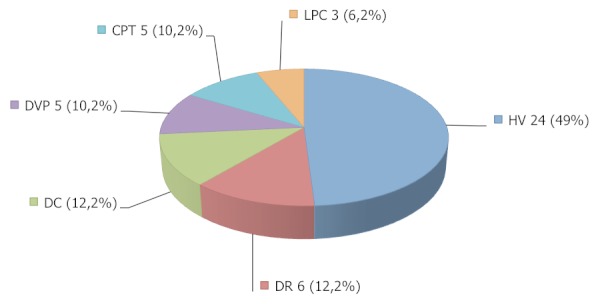
Fréquence des différentes lésions échographiques

**Figure 2:**
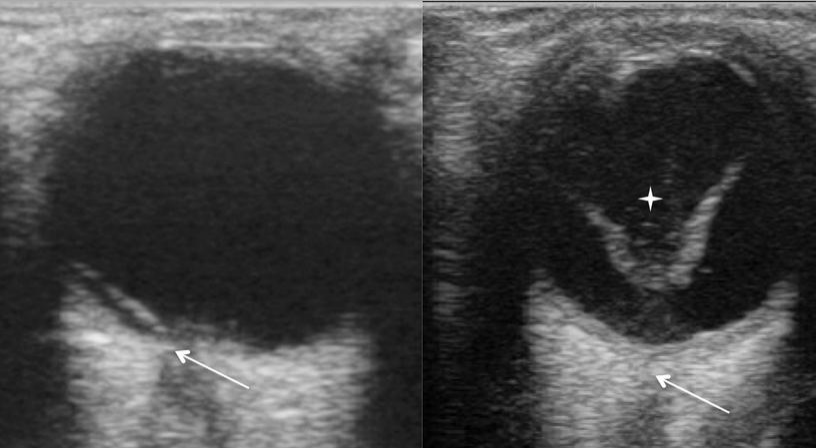
Images échographiques de décollements de la rétine. A) Décollement partiel avec attache papillaire (flèche); B) Décollement total avec une rétine épaissie rattachée en V à la papille (flèche) associé à une hémorragie intra-vitréenne se traduisant par des échos intra-vitréens (étoile à 4 branches)

L’échographie réalisée sur les 39 yeux traumatisés était anormale dans 38 cas (97,4%). Les lésions au nombre de 49 étaient multiformes et associées. Les différents diagnostics échographiques sont rapportés dans le Tableau 1 et la Figure 1 résume la fréquence des différentes lésions objectivées à l’échographie. Tous les cas de décollement choroïdien étaient accompagnés d’une hémorragie sous choroïdienne. La Figure 2 représente une image échographique de décollement de la rétine.

**Tableau 1:** Fréquence des différents diagnostics échographiques.

	Effectif	%
Hémorragie du vitré isolée (HV)	13	33,3
Décollement de la rétine (DR)	02	05,1
Décollement de la rétine + Hémorragie du vitré	04	10,2
Décollement de la choroïde	02	05,1
Décollement de la choroïde + Hémorragie du vitré	04	10,2
Décollement postérieur du vitré	02	05,1
Décollement postérieur du vitré + Hémorragie du vitré	03	07,7
Luxation postérieure du cristallin	03	07,7
Cataracte post traumatique	05	12,9

**Figure 3:**
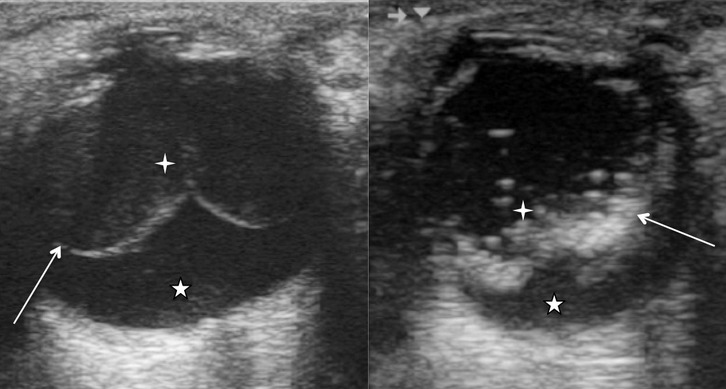
Images échographiques de décollements de la choroïde. A) Décollement de la choroïde (flèche) réalisant une image sans attache avec la papille avec hémorragie intra-vitréenne (étoile à 4 branches) et sous choroïdienne (étoile à 5 branches); B) Décollement de choroïde (flèche) avec hémorragie et prolifération vitréenne (étoile à 4 branches) et hémorragie sous choroïdienne (étoile à 5 branches)

**Figure 4:**
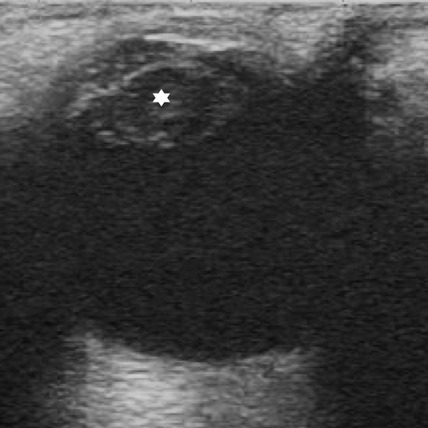
Image échographique d’une cataracte post traumatique

La Figure 3 montre une image échographique de décollements de la choroïde et la Figure 4 celle d’une cataracte post traumatique.

## Discussion

Le traumatisme oculaire est une affection relativement fréquente avec des conséquences graves car pouvant entrainer une cécité [1, 2]. Ces traumatismes s’accompagnent souvent d’une opacification des milieux transparents de l’œil rendant l’examen du fond d’œil peu contributif [4]; d’où la place de l’échographie en mode B qui permet dans ces cas de retrouver une étiologie organique aux baisses de l’acuité visuelle, ce qui a été le cas dans notre étude (97,4%).

Sur le plan épidémiologique, ces traumatismes oculaires surviennent préférentiellement chez l’adulte jeune comme dans notre série (29 ans). Ce constat est classique dans la littérature avec des moyennes d’âge allant de 26 à 29 ans [1, 2, 11, 12].

Le sexe masculin était prédominant dans notre série (62,5%). Ceci est corroboré dans d’autres séries avec des taux variables allant de 67,8% à 83,6%. [1, 2, 11, 13].

Les circonstances de survenue observée dans la littérature sont variables mais similaires d’une étude à l’autre avec cependant une fréquence plus élevée des AVP dans les pays Africains [1–3, 13].

Les traumatismes oculaires sont classiquement unilatéraux comme dans notre série (78,1%). Les contusions sont la variété anatomo-clinique la plus fréquente et notées dans l’intervalle de 67,5% à 82,85% [1–3] en concordance avec les 82% de notre série.

Dans notre étude, la lésion la plus fréquemment trouvée à l’échographie était une hémorragie du vitré (HV) dans 63% des cas. Plusieurs auteurs ont également signalé l’HV comme première anomalie échographique objectivée dans les traumatismes oculaires [11, 14]. Makosso dans une étude portant sur les baisses brutales de l’acuité visuelle quel qu’en soit l’étiologie, a rapporté que l’HV était également le premier diagnostic échographique (37,5%) [9]. Le diagnostic échographique est aisé en montrant des échogénicités en amas du vitrée. La diminution de transparence des milieux qu’elles entrainent justifie la réalisation systématique d’une échographie oculaire lorsque le diagnostic clinique de cette lésion est réalisée [4, 7, 15]. Cette hémorragie est soit isolée ou associée à d’autres pathologies. La principale pathologie associée à une HV est le décollement de la rétine (DR) [7, 14]. Ce décollement peut survenir à distance du traumatisme, ce qui justifie une surveillance échographique des HV [4].

Le DR retrouvée dans 15% des cas dans notre série fait la gravité des traumatismes oculaires. [16] Ce surtout à Parkou où tous les moyens thérapeutiques ne sont pas disponibles. Dans la littérature, cette lésion est observée dans des proportions variables allant de 26,89% à 62,5%. [11, 13, 14, 17]. L’échographie a une bonne sensibilité dans le détection des DR allant de 97% [18], à 100% [10]. Les déchirures rétiniennes sont quant à elles de diagnostic difficile en dehors des déchirures géantes. En effet, d’après Rabinowitz [7] seul 44% des cas sont correctement diagnostiqués à l’échographie. Ceci pourrait expliquer l’absence de cette lésion dans notre série.

Le décollement de la choroïde (DC) est rapporté par plusieurs auteurs comme le second type de décollement de membrane [5, 7, 14]. Dans notre étude, nous avons observé un nombre identique de DR et de DC.

Un décollement postérieur du vitré (DPV) était objectivé dans 10,2% des cas dans notre série. Cette anomalie a été objectivée dans 20% des cas dans l’étude de [5] et dans 11% des cas dans l’étude de [14].

La cataracte post traumatique observée dans une proportion de 12,8% dans notre série est une entité fréquente signalée dans des proportions de 18,3% dans l′étude de Kwong et al. [4] et dans 19,4% des cas dans celle d’Eze [13]. La luxation postérieure du cristallin, lésion dû à un choc direct était observée dans 6,2% des cas et dans 8,9% des cas par [13].

## Conclusion

L’échographie oculaire est un examen simple, non irradiant qui même réalisé avec un appareil polyvalent permet un bilan lésionnel satisfaisant des traumatismes oculaires.
